# Effects of Light on Secondary Metabolites in Selected Leafy Greens: A Review

**DOI:** 10.3389/fpls.2020.00497

**Published:** 2020-04-24

**Authors:** Felix Thoma, Annette Somborn-Schulz, Dennis Schlehuber, Volkmar Keuter, Görge Deerberg

**Affiliations:** Fraunhofer Institute for Environmental, Safety, and Energy Technologies UMSICHT, Oberhausen, Germany

**Keywords:** leafy green, secondary metabolite, flavonol, anthocyanin, carotenoid, light, photoreceptor

## Abstract

In contrast to the primary metabolism, responsible for essential synthesis mechanisms and mass balance in plants, the secondary metabolism is not of particular importance for each cell but for the plant organism as its whole. Most of these metabolites show antioxidant properties and are beneficial for human health. In order to affect accumulation of those metabolites, light is an essential factor. It is possible to select various combinations of light intensity and light quality to address corresponding photoreceptors and synthesis. However, the plethora of additional variables considering environmental conditions such as temperature, relative humidity or cultivation method complicate defining specific “light recipes”. This review summarizes experiments dealing with consumable leafy greens such as lettuce or basil and the enhancement of three selected metabolites – anthocyanins, carotenoids and flavonols.

## Introduction

Light greatly affects the biosynthesis and accumulation of various secondary plant metabolites that are crucial for crop quality (Siddiqui and Prasad, 2017). In contrast to primary metabolites, secondary metabolites such as anthocyanins, carotenoids or flavonols are minor compounds in plants occurring in low concentrations. They are not essential for life but play a major role in the plants fitness for survival (Pagare et al., 2015) and occur in most fruits and vegetables (Brglez Mojzer et al., 2016). Typical functions are cell pigmentation in order to attract pollinators and seed dispersers or protection against UV radiation or other abiotic and biotic stresses (Samanta et al., 2011; Mosadegh et al., 2018). In humans, they display various beneficial health effects. Due to their antioxidant activity, many of them show anti-microbial, anti-inflammatory and anti-allergic effects and are able to prevent diseases (Pedro et al., 2016; Lu et al., 2017).

**FIGURE 1:**
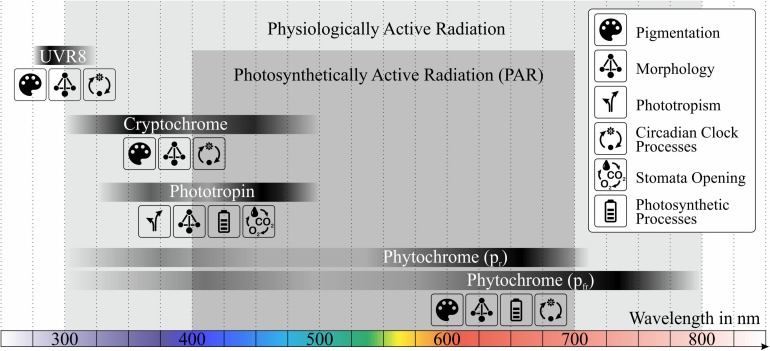
Absorption bands of photoreceptors in plants and their affected quantities or processes, data obtained from Casal (2000), Schopfer and Brennicke (2010), and Folta and Carvalho (2015). In order to address secondary metabolites in plants, photosynthetically active radiation (PAR) is not sufficient, but a wider wavelength range (physiologically active radiation) must be considered.

The biosynthesis and accumulation of those secondary metabolites is mainly triggered through light. Photoreceptors are linked to signaling pathways and lead to gene expression changes when being activated by photons. The combination of a photoreceptor protein and a chromophore defines the light absorbing properties (Tilbrook et al., 2013; Folta and Carvalho, 2015). Absorption bands of essential photoreceptors and their affected quantities and processes are shown in Figure 1. In general, only a small fraction of absorbed photons is used for activating photoreceptors compared to those used for photosynthesis (Schopfer and Brennicke, 2010).

There are three main variables considering light requirements in horticulture: light quality, light quantity and photoperiodism (Kozai et al., 2016). In terms of light quality, various spectral composition having either narrow peaks or a smooth broadband spectrum can be generated with either LED fixtures, HPSL (high pressure sodium lamp) or other light sources. Regarding light quantity, modern light sources are able to exceed the light saturation point (PPFD at which net photosynthetic rate per unit leaf area becomes maximum) in leafy greens, which is in general less than 1000 μmol/m^2^/s (Kozai et al., 2016). In terms of photoperoidism, not only the ratio of day and night period, but also supplemental exposure periods are possible. With these three variables the amount of realizable light settings is almost infinite.

When talking about lighting in horticulture, it is common to use units adapted to plants and photosynthesis. PAR (photosynthetically active radiation) is responsible for photosynthesis in plants and is defined as the wavelength range between 400 and 700 nm (Figure 1). The PPFD (photosynthetic photon flux density) is the integral of the irradiation E from 400 to 700 nm weighted with the wavelength λ and a factor.

$$P\,P\,F\,D=\int_{400}^{700}E_{\lambda }\times \lambda \times 0.00836\,d\lambda$$

The factor results from the unit μmol/m^2^/s and its connection to the Avogadro constant, which indicates that not the energy of photons but the number of photons is crucial (McCree, 1972; Tazawa, 1999).

Besides radiation between 400 and 700 nm, also photons with lower or higher wavelengths contribute partly to photosynthesis, morphologic processes or the synthesis of secondary metabolites (Tazawa, 1999). For those physiological processes, the wavelength range between 300 and 800 nm is defined as the less well-known physiologically active radiation (see Figure 1). Those definitions should be handled with care since UV light for example is not included in the PAR domain and does not contribute to the total PPFD but can still be stated in μmol/m^2^/s.

Additionally to the three light variables mentioned above (light quality, light quantity and photoperiodism), further environmental grow parameters, such as temperature, relative humidity or irrigation are variable and require specific settings depending on plant species and cultivars. The amount of variables opens a large parametric space, which complicates a comparability of studies and experiments regarding the influence of light on the accumulation of secondary metabolites. This review summarizes the results of various studies with the aim of showing trends and parallels regarding the effects of certain light scenarios on the accumulation of three selected secondary metabolites in leafy greens. For this purpose, articles containing “leafy,” “microgreen,” “salad,” “lettuce,” “basil,” “spinach,” “parsley,” “rocket,” “chard” or “lovage” were considered.

## Photoreceptor Proteins

The following section gives an overview on the mechanisms and main tasks of the four major photoreceptors in plants and their absorption bands (see Figure 1). These receptors absorb photons not only in the PAR domain, but also wavelengths in the UV and far-red region. They interact with further signal transduction elements and are responsible for triggering different processes, such as the biosynthesis of secondary metabolites (Frankhauser and Chory, 1997).

### Cryptochrome

The absorption of the protein cryptochrome peaks in the UV-A and blue light domain between 340 and 520 nm (Figure 1; Schopfer and Brennicke, 2010). Due to the non-covalent bond of cry to one of two possible chromophores, two absorption maxima exist. The first maximum at 375 nm results from the chromophore 5,10-methenyltetrahydrofolic acid (MTHF), the second at 450 nm from the chromophore flavin [in the form of flavin adenine dinucleotide (FAD)] (Fuller et al., 2016). Dependent on transient redox state, the flavin chromophore shows different absorption properties. When being exposed to blue light, it shifts to a semi-reduced form with its absorption in the green and yellow domain. Being then illuminated with green or yellow light, the cryptochrome gets inactivated (Folta and Maruhnich, 2007). It was shown, that green light is able to reverse the effects of blue light, such as anthocyanin accumulation, inhibition of extension growth or stimulation of stomata opening (Folta, 2004; Bouly et al., 2007; Wang and Folta, 2013). Additionally, flowering is inhibited, which is known to be induced by blue light, mainly through cry2 (Folta and Carvalho, 2015). Besides morphologic aspects, such as plant or root growth, fruit size or stem elongation, cryptochrome is responsible for regulating processes linked to the circadian clock, like seedling development, guard cell opening, photoperiodic flowering control or de-etiolation (greening after period of darkness) (Ahmad and Cashmore, 1993; Guo et al., 1998; Devlin, 2000; Folta and Spalding, 2001; Mao et al., 2005; Fox et al., 2012). The use of knock-out mutants regarding cryptochrome in plants restricts mainly on Arabidopsis (Kang and Ni, 2006; Popov et al., 2010). Barrero et al. (2014) demonstrated by using a knockdown of cry1a/b and cry2 mRNA, that cry1 is responsible for grain germination in barley. A rough separation with other blue light receptors can also be made by changing the light intensity, since cryptochrome tends to have high-fluence-rate responses (Folta and Carvalho, 2015). Due to lower levels of blue light below canopies cryptochrome also shows a phototropic response (Goyal et al., 2016). Additionally, Fox et al. (2012) showed that the accumulation of anthocyanins is controlled by cryptochrome.

### Phytochrome

Phytochrome, a hydrophilic protein, is responsible for the absorption of light mainly in the red/far-red region around 665 nm (P_r_) and 730 nm (P_fr_), but also in blue/near-UV region (Figure 1). It shows a large range in sensitivity (from moonlight to full sunlight) and controls morphologic and physiologic parameters, but also pigmentation. phyA and phyB are the best-known phytochromes, however, stability inside the cell and functionality differ between them. For both types, P_r_ is generated in darkness and converts reversible to P_fr_ when illuminated (Deutsches Institut für Normung, 2018). When light stimulates phytochrome, events in cytoplasm and nucleus are initiated. Many of them are related to alterations in hormone levels such as gibberellins and auxins (plant hormones concerning growth), ethylene, jasmonates (plant hormone concerning growth and photosynthesis) and abscisic acid (ABA) (plant hormone concerning development, control of organ size, stomatal closure) (Casal, 2000; Schopfer and Brennicke, 2010).

### Phototropin

The protein phototropin absorbs light in the range between 340 and 520 nm (Figure 1) and is mainly responsible for a direct or indirect optimization of photosynthesis (e.g. phototactic orientation of chloroplasts) (Schopfer and Brennicke, 2010). phot1 acts under low light intensity (leaf expansion/position) and phot2 is activated and acts redundantly with phot1 under higher light intensities (Takemiya et al., 2005). As the name implies, phototropin is responsible for phototropism and the optimization of plant growth and development for better photosynthesis. Especially through low fluence in the blue light domain, phototropin suppresses leaf curling (promoted by phyB), which results in flatter leaves and thus a higher light absorption (Folta and Carvalho, 2015). Additionally, phototropin partially controls the opening of stomata und thus transpiration (Schopfer and Brennicke, 2010).

### UVR8

Considering the UV range from 280 to 350 nm, the protein UVR8 shows maximum absorption (Figure 1; Schopfer and Brennicke, 2010). Since UV-radiation – especially at short wavelengths – is able to damage DNA or proteins by the generation of reactive oxygen species (ROS), plants have certain damage-repair mechanisms and UV absorbing pigments. UVR8 generates gene expressions that induce the accumulation of such protective pigments (e.g. anthocyanins). Furthermore, UVR8 influences plant architecture in a way that plants grow more compact concerning stem elongation and leaf expansion. UVR8 enables producers the control of growth without photosynthetic radiation (400–700 nm) (Casal, 2000; Cope and Bugbee, 2013; Coffey et al., 2017).

## Effects of Light on Secondary Metabolites

The following sections states the effect of light on three representative groups of secondary metabolites – anthocyanins, carotenoids and flavonols. Tables 1–3 summarizes those studies regarding fixed parameters, variable light parameters and their qualitative effect on the investigated metabolite in selected plants.

**TABLE 1:** Relative accumulation of anthocyanins after different treatments.

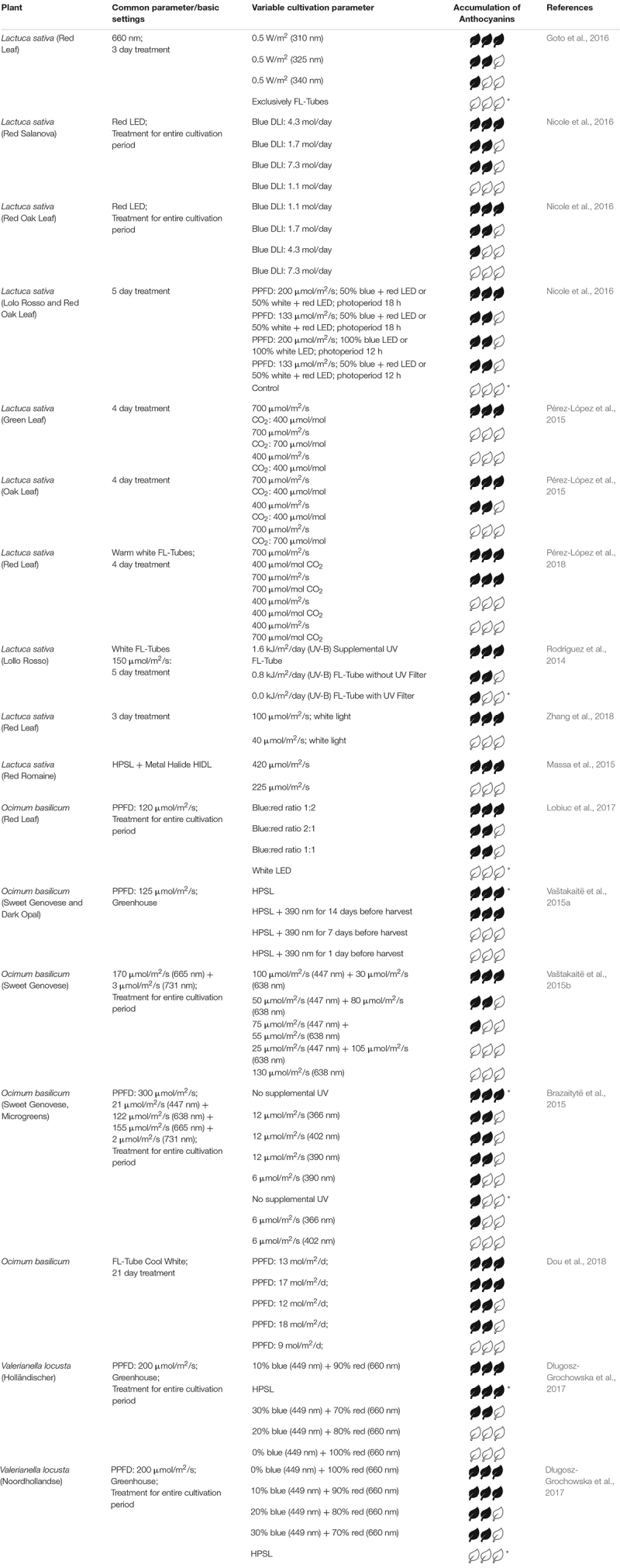

*Three filled leaves indicate the highest value within one study, three unfilled leaves the lowest one. The scale is a quantitative one. HPSL: High Pressure Sodium Lamp; FL-Tube: Fluorescent Tube; PPFD: Total Photosynthetic Photon Flux Density; HIDL: High Intensity Discharge Lamp; DLI: Day Light Integral. The asterisk indicates the control group, if available.*

**TABLE 2:** Relative accumulation of carotenoids after different treatments.

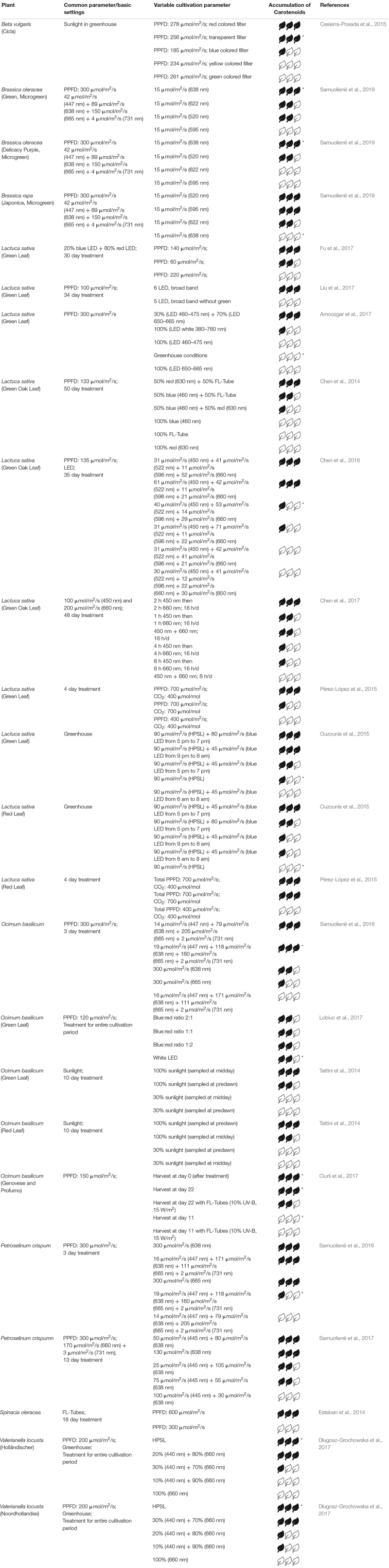

*Three filled leaves indicate the highest value within one study, three unfilled leaves the lowest one. The scale is a quantitative one. HPSL: High Pressure Sodium Lamp; FL-Tube: Fluorescent Tube; PPFD: Total Photosynthetic Photon Flux Density; HIDL: High Intensity Discharge Lamp; DLI: Day Light Integral. The asterisk indicates the control group, if available.*

**TABLE 3:** Relative accumulation of flavonols after different treatments.

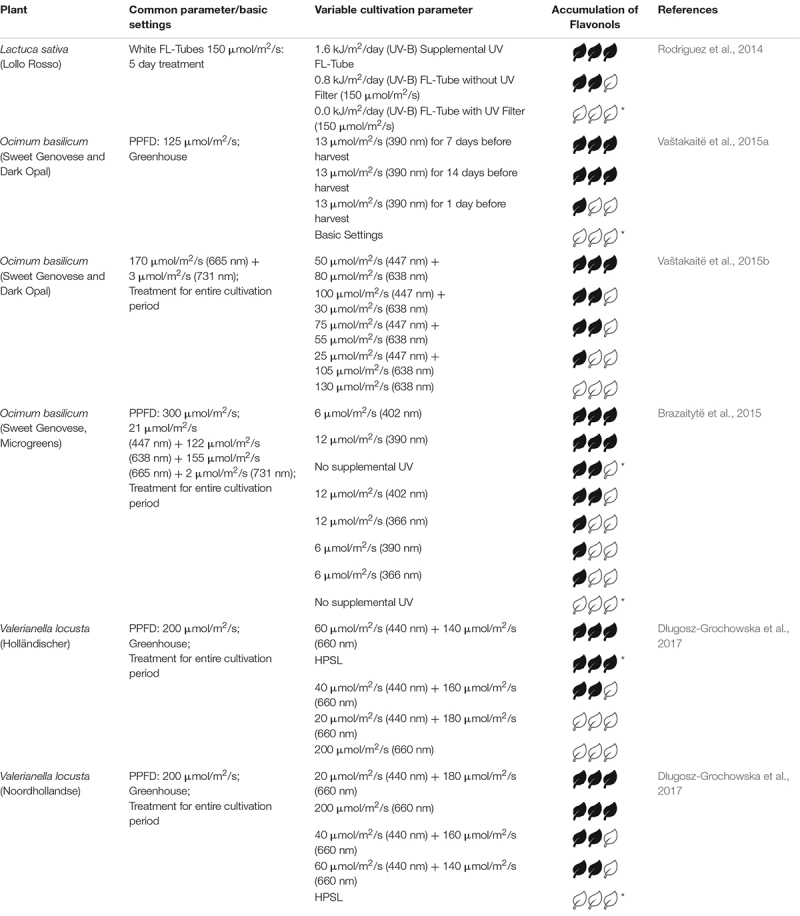

*Three filled leaves indicate the highest value within one study, three unfilled leaves the lowest one. The scale is a quantitative one. HPSL: High Pressure Sodium Lamp; FL-Tube: Fluorescent Tube; PPFD: Total Photosynthetic Photon Flux Density; HIDL: High Intensity Discharge Lamp; DLI: Day Light Integral. The asterisk indicates the control group, if available.*

### Anthocyanins

So far, 35 different anthocyanidins and more than 600 anthocyanins (glycoside form of anthocyanidins) are known. Six anthocyanidins (cyanidin 50%, delphinidin 12%, pelargonidin 12%, peonidin 7%, petunidin 7% and malvidin 7%) are common in plants (Kong et al., 2003; Andersen and Jordheim, 2010). The glycoside derivatives of the three non-methylated anthocyanidins (cyanidin, delphinidin and pelargonidin) are found in 80% of pigmented leaves, 69% in fruits and 50% in flowers (Evans, 1991). Additionally, grains and roots can contain anthocyanins (Khoo et al., 2017). Produced in the cytoplasm, anthocyanin molecules accumulate in the vacuole, where they are solved in the cell sap (Schopfer and Brennicke, 2010). They show effects on growth and feeding behavior of insects, protect the plant against UV radiation or act as attractor to animals in order to spread seeds and pollen (Onyilagha et al., 2004; Khalid et al., 2019). Due to their ability to donate a hydrogen atom (oxidation) e.g. to reactive free radicals, anthocyanins are decent antioxidants (Castañeda-Ovando et al., 2009). They have shown a higher antioxidant activity than vitamins C and E (Bagchi et al., 1998). Further health benefits of anthocyanins are antiangiogenesis, prevention of CVD, anticancer, antidiabetes, improved visual health, anti-obesity, antimicrobial, and neuroprotection (Khoo et al., 2017; Khalid et al., 2019). In general, anthocyanins show a low bioavailability, which means only a small amount is absorbed into the blood cycle during digestion. Khoo et al. (2017) also summarize that increased consumption may enhance their efficacy, however, side effects of overconsumption are not investigated yet. In 2001, Bieza and Lois (2001) reported a protecting effect of anthocyanins in *Arabidopsis uvt1* mutants (hypersensitive to UV) against UV radiation. The extract of UV irradiated leaves absorbs more light between 300 and 400 nm than the non-irradiated one. The exposure induces the development of UV protecting anthocyanins (Bieza and Lois, 2001).

In their review from 2015, Bian et al. (2015) summarize that especially UV light optimizes the accumulation of phenolic compounds in general. In particular, the concentration of anthocyanins is affected by mainly blue, UV-A and UV-B light. Considering *Lactuca sativa* (green leaf), Rodriguez et al. (2014) found that the use of UV Fluorescent-Tubes (FL-Tubes) supplementary to sunlight results in a maximum of anthocyanins content. Similarly, Goto et al. (2016) obtained an increase in anthocyanins exposing red leaf lettuce to three different UV wavelengths in the order 310 nm >325 nm >340 nm. In contrast to that, Vaštakaitë et al. (2015a) showed that anthocyanin content in basil was highest without UV radiation. Similarly, exposing basil to three supplementary UV wavelengths at two intensities, the group of Brazaitytë et al. (2015) found highest content of anthocyanins in the control group.

Since not only UVR8 and cryptochrome but also low amounts of either phyA or phyB are relevant for the accumulation of anthocyanins, wavelengths beyond UV also show effects. Considering the effect of blue light, the anthocyanin index was dependent on variety, even between different red lettuce varieties. Red Oak lettuce showed maximum values at highest daily blue light integral (BDLI at 7.3 mol/day) and for Red Salanova lettuce the maximum anthocyanin index occurred at a BDLI of 4.3 mol/day. Both varieties had its minimum at the lowest BDLI of 1.1 mol/day (Nicole et al., 2016). A blue:red ratio of 1:2 for *Ocimum basilicum* showed best results compared to other ratios (Lobiuc et al., 2017). Similar results obtained the group of Vaštakait, but using two red channels (Vaštakaitë et al., 2015b). For two cultivars of *Valerianella locusta* the maximum accumulation of anthocyanins was determined at a blue:red ratio of 1:9 and only red light. However, for one cultivar the control group under HPSL also showed maximum values whereas the control group of the second cultivar had the lowest content of anthocyanins (Długosz-Grochowska et al., 2017). Far-red light reduces the synthesis of anthocyanins (Bian et al., 2015).

Besides light quality, light intensity and photoperiodism where investigated in various studies. In general, a light intensity between 200 and 300 μmol/m^2^/s is suitable for plants grown in controlled environments (Bian et al., 2015). In two studies Pérez-López et al. (2015, 2018) showed that the combination of a high light intensity and low CO_2_ values is a good condition for the accumulation of anthocyanins not only in green but also in red leaf lettuce. Nicole et al. (2016) increased the anthocyanin index in Red Oak and Red Salanova lettuce having a long photoperiod of 18 h a day compared to 12 h a day and a PPFD of 200 μmol/m^2^/s. Both varieties showed similar behavior considering changes in photoperiodism and DLI (day light integral) differing only in absolute anthocyanin index values. Under cool white FL-Tubes the anthocyanin content of basil was highest at a DLI of 13 and 17 mol/m^2^/d (Dou et al., 2018).

In general one can say that supplementary UV-A and UV-B light induces the synthesis of anthocyanins in many leafy greens, especially in red leafy greens. One exception is basil, where the addition of UV light and the accumulation of anthocyanins correlates negatively. A blue:red ratio between 1:1.5 and 1:2 serves as a reliable basis concerning the accumulation of anthocyanins in leafy greens. Comparing studies dealing with basil, similarities in applying a low blue:red ratio as optimal light condition can be observed. However, Vaštakaitë et al. (2015b) showed, that no blue light at all resulted in low anthocyanin levels (Lobiuc et al., 2017). For *Valerianella locusta* (var. Holländischer), similar effects were observed, whereas var. Noordhollandse showed highest contents without blue light (Długosz-Grochowska et al., 2017). This indicates that even within different varieties optimal light conditions differ. For detailed information see Table 1.

### Carotenoids

Carotenoids fulfill two major functions: on the one hand, they harvest light and protect the plant from high exposures (xanthophyll cycle), and on the other hand, they attract animals in order to spread the plants seeds and pollen. Carotenoids include two major sub-groups: carotenes (α-carotene, β-carotene, lycopene) and xanthophylls (lutein, zeaxanthin, etc.). Both are located in the thylakoid membrane but in contrast to xanthophylls, carotenes contain no oxygen. Since synthesis of carotenoids is absent in animals, the only way for humans to ingest them is via plants (Adams et al., 1995; Schopfer and Brennicke, 2010; Bian et al., 2015). The human body is able to convert α-carotene, β-carotene, and β-cryptoxanthin, each being a provitamin A, into retinol. Retinol has the same action spectrum as vitamin A (Higdon, 2004). Grodstein et al. (2007) showed in a long-term study that the supplemental β-carotene every second day reduced cognitive decline in over 4052 male participants. Regarding human vision, Kvansakul et al. (2006) showed that a supplementation of lutein or zeaxanthin improved contrast acuity thresholds at high mesopic levels (dim light situations). According to Gallicchio et al. (2008) there is only a small and not statistically significant association between β-carotene and the decrease in development of lung cancer. However, carotenoids in general exhibit antioxidant properties (Esteban et al., 2014).

Regarding the carotenoid concentration in plants, Bian et al. (2015) summarize that particularly blue, red and UV-B light affect the accumulation of carotenoids in vegetables. Brazaitytë et al. showed that supplemental UV or blue light (366, 390 or 402 nm) decreases the carotenoid content in basil. Similarly, Ciurli et al. (2017) ascertained, that supplemental UV light from UV FL-Tubes does not promote accumulation of carotenoids.

In 2017, Liu et al. (2017) observed a slightly higher carotenoid accumulation in *Lactuca sativa* when adding green light and keeping the total PPFD constant. Exposing green leaf lettuce to 50% red LED light and 50% white light from FL-Tubes yielded a higher content of carotenoids than red and blue LED light (1:1) or sole FL-Tube light. With the use of white LEDs plus monochromatic LEDs, the same group obtained a maximum carotenoid concentration by using white plus red or white plus blue LEDs (Chen et al., 2016). For green leaf basil, Lobiuc et al. (2017) identified no significant change when altering the blue:red ratio concerning carotenoid accumulation. Samuolienë et al. (2016) exposed basil to blue and red LED light and investigated carotenoid content. They found it to be maximum when the amount of blue light (447 nm) was low compared to red light and the intensity at 665 nm was higher than at 638 nm. The results of Brazaitytë et al. (2016) showed, that the addition of 15 μmol/m^2^/s of green LED light (520 nm) increased the amount of carotenoids. Besides HPL, a blue:red ratio of 1:4 or 3:7 generated maximum accumulation of carotenoids in two cultivars of lamb’s lettuce (Długosz-Grochowska et al., 2017). In contrast to that, Amoozgar et al. (2017) obtained a significant increase of carotenoids in lettuce using only blue and red light (3:7) compared to white LED light at a constant total PPFD. Exposing various microgreens (Amaranth, Cress, Mizuna and Purslane) to either sole blue or red light resulted in low carotenoid levels compared to applying both wavelengths together (Kyriacou et al., 2019). The group of Casierra-Posada found out that the highest amount of carotenoids in *Beta vulgaris* was obtained by implementing red colored or transparent filters in a greenhouse. However, blue or red colored filters also caused a lower total PPFD (Casierra-Posada et al., 2015). Illuminating three different *Brassicaceae* microgreens with supplemental green, yellow or orange light, a promoting effect regarding accumulation of carotenoids was only observed for mizuna. For broccoli and kohlrabi the control group displayed highest carotenoid levels (Samuolienë et al., 2019).

Keeping the blue:red ratio constant at 1:4 and altering the total PPFD, the maximum content of carotenoids in lettuce was found at 140 μmol/m^2^/s (Fu et al., 2017). Considering red and green lettuce, Ouzounis et al. (2015) showed maximum carotenoid concentration by supplementing daylight with blue LEDs (80 μmol/m^2^/s) and HPSL (90 μmol/m^2^/s) for 2 h in the evening.

According to the studies mentioned above, UV light does not promote the synthesis of carotenoids. In contrast to that, supplementary wavelengths in the green domain increase carotenoid accumulation, independent of light source (HPSL, FL tube or white LED). For green leaf lettuce this was shown e. g. with red LEDs plus FL tubes (Chen et al., 2014), however, the group of Liu et al. (2017) obtained decreased carotenoid content after illuminating lettuce with supplementary green light. Considering the optimal blue to red ratio for lettuce, Chen et al. (2016) obtained best results with a ratio of either 0.6 or 3. Nevertheless, the majority of studies work with a blue:red ratio below 0.5 (Amoozgar et al., 2017; Fu et al., 2017). For basil, the ratio depends on variety. For detailed information see Table 2.

### Flavonols

Besides quercetin and its glycoside rutin, kaempferol and myricetin are the most common flavonols in plants. Similar to carotenoids, flavonols show antioxidant properties (Böhm et al., 1998; Graf et al., 2005). Since they exhibit a strong absorbance in the short wavelength domain, they protect DNA and other UV sensible molecules against UV radiation (Bilger et al., 2001; Merzlyak et al., 2005; Rodriguez et al., 2014). Flavonols not only protect the plant against abiotic stresses (UV radiation, drought or heat) but also biotic stresses, such as herbivore and pathogen attack (Khalid et al., 2019). Compared to anthocyanins, flavonols are in general more stable molecules. This can be observed for example at the occasional color fading of strawberries (Böhm et al., 1998). Besides onions, broccoli or apples, various leafy greens contain flavonols (Graf et al., 2005; Vaštakaitë et al., 2015b; Długosz-Grochowska et al., 2017). Similar to anthocyanins, in humans they display various health benefits due to their ability of scavenging free radicals (Khalid et al., 2019).

Rodriguez et al. (2014) determined an increased concentration of flavonols in lettuce using supplemental UV FL-Tubes additional to sunlight and supplemental HPSL. Similarly, basil was exhibiting a higher flavonol content when being illuminated with 13 μmol/m^2^/s at 390 nm for 7 and 14 days before harvest (Vaštakaitë et al., 2015a). Brazaitytë et al. (2015) found out that supplemental 6 or 12 μmol/m^2^/s at a wavelength of 402 or 390 nm, respectively, produced best results regarding flavonol index. Exposing basil to constant 170 μmol/m^2^/s at 665 nm and adjustable intensities at 447 and 638 nm, the intensity providing best results was 50 and 80 μmol/m^2^/s, respectively (Vaštakaitë et al., 2015b).

In contrast, Długosz-Grochowska et al. (2017) obtained different results using the same wavelength and ratios for two cultivars of lamb’s lettuce. For the first cultivar, illumination with HPSL or a ratio of blue to red of 3:7 showed best effects. The second one had maximum flavonol content at a blue:red ratio of 1:9 or no blue light and minimum content after illumination with HPSL (Długosz-Grochowska et al., 2017).

The concentration of both flavonols, quercetin and its glycoside rutin, were investigated in basil with supplemental UV-B irradiation. Best results for both were obtained at an irradiance of 3.6 W/m^2^ for a photoperiod of 8 h. For 10 h exposure, minimal rutin and quercetin content were detected (Ghasemzadeh et al., 2016). The rutin content in lamb’s lettuce was shown to be highest after applying a blue:red ratio of 1:1. Keeping the total PPFD constant, less blue light, HPSL, cold or warm white LEDs yielded lower contents (Długosz-Grochowska et al., 2016).

The underlying blue to red ratio considering flavonoid accumulation is species dependent and lies in the range between 1:1 and no blue light at all. A promoting factor for accumulation of flavonols in leafy greens is supplementary light in the UV range. In contrast to anthocyanins and carotenoids accumulation, basil shows a positive correlation regarding supplementary UV light and the accumulation of flavonols. In total, the applied PPFD for most leafy greens is in the range of 150 – 300 μmol/m^2^/s. For detailed information see Table 3.

## Discussion

The present work gives an overview of the latest results and reveals tendencies concerning the accumulation of selected secondary metabolites triggered through light. In order to address corresponding photoreceptors, specific wavelength bands must be provided. Due to its monochromatic characteristics, its rapid advancement and consequent cost reduction, LED technology is well suited for conducting precise cultivation experiments and later on cultivation conditions.

When comparing optimal light conditions regarding the accumulation of anthocyanins between those for carotenoids or flavonols, we conclude blue:red ratios being smaller than 1 to be optimal. For carotenoids and especially for flavonols, the amount of blue light can decrease to 0 – depending on the specific type of phenolic compound and plant species. In contrast to anthocyanins, supplementary green light promotes the accumulation of carotenoids in most of leafy greens. This coincides with their absorption maximum in the green light domain. In general, for all three secondary metabolites UV light is a promoting factor, except for the accumulation of anthocyanins in basil.

Since the aim often is to increase nutritional value and quality of crops, not only levels of certain secondary metabolites but also their biological activity is an important parameter to investigate. Studies, which investigated for example the free radial scavenging activity, in general showed a positive correlation with levels of either flavonoids or carotenoids. For basil, Samuolienë et al. (2016) found an increased ability of scavenging free radicals when applying either a relatively high or low ratio of 638 to 665 nm. Red light increased the free radical scavenging activity in green basil and blue light in red basil cultivars (Lobiuc et al., 2017). Within two varieties of *Valerianella locusta* the free radical scavenging activity differed under different light conditions. For Holländischer, increased activity was detected after supplementary blue light, for Noordhollandse no significant changes were measured, except for illumination with relatively high red light intensities (Długosz-Grochowska et al., 2017). Supplementary far-red light or an increased blue:red ratio enhances antioxidant activity in green leaf and red leaf lettuce, respectively (Son and Oh, 2015; Lee et al., 2016).

Besides redundant and overlapping results, in many cases a comparison between multiple studies is challenging. This is mainly due to different experimental setups considering not only light conditions but also other environmental parameters or plant varieties. Furthermore, the use and mixture of different units, such as W/m^2^, μmol/m^2^/s or % complicate comparability. In order to increase comparability, more uniform standards regarding cultivation conditions are required. Consequently, future work will help to detect further correlations between light and the accumulation of secondary metabolites or their biological activity with increasing certainty. Those specifically tailored light spectra will enable growers to produce high quality crops not only for human nutrition but also for medicinal purposes in an efficient manner. In order to be flexible regarding cultivar variety and having the ability to grow products of high nutritional value, producers need to invest in adjustable light fixtures, which cover more than PAR domain.

## Author Contributions

FT conceptualized and designed the work, collected, analyzed, and interpreted the data, drafted the manuscript. AS-S and DS collected the data, contributed to critical revision of the manuscript. VK and GD contributed to critical revision of the manuscript, approved the final version to be published.

## Conflict of Interest

The authors declare that the research was conducted in the absence of any commercial or financial relationships that could be construed as a potential conflict of interest.
